# Hardware-Efficient Stochastic Binary CNN Architectures for Near-Sensor Computing

**DOI:** 10.3389/fnins.2021.781786

**Published:** 2022-01-05

**Authors:** Vivek Parmar, Bogdan Penkovsky, Damien Querlioz, Manan Suri

**Affiliations:** ^1^Department of Electrical Engineering, Indian Institute of Technology Delhi, New Delhi, India; ^2^Centre de Nanosciences et de Nanotechnologies, Université Paris-Saclay, CNRS, Palaiseau, France

**Keywords:** stochastic computing (SC), binarized neural network (BNN), RRAM (resistive RAM), in-memory computing (IMC), near-sensor computing

## Abstract

With recent advances in the field of artificial intelligence (AI) such as binarized neural networks (BNNs), a wide variety of vision applications with energy-optimized implementations have become possible at the edge. Such networks have the first layer implemented with high precision, which poses a challenge in deploying a uniform hardware mapping for the network implementation. Stochastic computing can allow conversion of such high-precision computations to a sequence of binarized operations while maintaining equivalent accuracy. In this work, we propose a fully binarized hardware-friendly computation engine based on stochastic computing as a proof of concept for vision applications involving multi-channel inputs. Stochastic sampling is performed by sampling from a non-uniform (normal) distribution based on analog hardware sources. We first validate the benefits of the proposed pipeline on the CIFAR-10 dataset. To further demonstrate its application for real-world scenarios, we present a case-study of microscopy image diagnostics for pathogen detection. We then evaluate benefits of implementing such a pipeline using OxRAM-based circuits for stochastic sampling as well as in-memory computing-based binarized multiplication. The proposed implementation is about 1,000 times more energy efficient compared to conventional floating-precision-based digital implementations, with memory savings of a factor of 45.

## 1. Introduction

Artificial intelligence (AI) and deep learning research have enabled innovative solutions for a wide variety of vision applications at the edge. As a result, there has been increasing focus on developing low-precision AI solutions while maintaining accuracy equivalent with floating-point precision (Moons et al., 2017). With the emergence of binarized neural networks (BNNs), it has become possible to map complex multiply-and-accumulate (MAC) operations to simple logic gates such as exclusive-NOR (XNOR) and population count (popcount) operations. This simplification leads to savings in energy, area and latency at the cost of a moderate loss in accuracy (Courbariaux et al., 2016).

For most hardware BNNs demonstrated in literature, the input layer is typically implemented either in floating-point or 8-bit integer (int8) precision, whereas the subsequent layers use binarized neurons. In order to map all operations to a truly binarized pipeline, the computation of input layer using stochastic sampling has been proposed (Lee et al., 2017). A fully binarized pipeline can allow executing computation at the edge, without relying on network communication with high-performance compute servers in order to perform floating-point computations (Zhou et al., 2019). To further improve energy efficiency of such technologies, near-sensor computing has been investigated (Conti et al., 2018; Plastiras et al., 2018). Recently, stochastic binary neural networks have been proposed for mono-channel convolutional neural networks (CNNs) (Lee et al., 2017; Hirtzlin et al., 2019) to enable near-sensor computing. Such networks can be used for performing first-level computations for applications such as remote sensing, material analysis, medical image analysis, and so on (Hsu et al., 2019; Zhou and Chai, 2020).

A significant challenge of stochastic computing approaches, however, is the generation of high-quality random numbers with a low energy budget. Stochastic sampling for implementing TRNG (True Random Number Generators) using analog properties of circuits and devices has been studied in literature in order to develop more secure as well as area-efficient circuits (Jiang et al., 2017; Sahay et al., 2017; Jerry et al., 2018; Qu et al., 2018; Guo et al., 2019; Park et al., 2019; Huang et al., 2020; Simion, 2020). However, in most cases a non-uniform distribution, i.e., normal or log-normal, has been observed. Such distributions are attractive for applications such as Bayesian learning (Lin et al., 2019; Malhotra et al., 2020), Monte Carlo sampling (Dalgaty et al., 2021), or deep Boltzmann machines (Parmar and Suri, 2020). Unfortunately, uniform distribution, which is typically used for stochastic computing, is normally obtained only by additional circuit overheads leading to increased costs in terms of area and energy (Gong et al., 2019).

In this paper, we propose a novel method for realizing stochastic binary neural networks for performing classification on RGB images. We first use the CIFAR-10 dataset for validation. Then, to demonstrate a real-world application, we use the proposed network for microscopy image analysis. Benefits of implementing the proposed network based on emerging OxRAM technology for both stochastic sampling as well as XNOR computation are also analyzed in detail.

Key contributions of this work are as follows:

- Stochastic sampling at input layer based on normal distribution is demonstrated for realizing stochastic binarized convolutional neural network (SBCNN) with validation over the CIFAR-10 dataset.
- The first demonstration of stochastic BNNs for microscopy application, matching reported accuracy from literature with large memory savings (≈45 ×) and energy savings (≈1000 ×) compared to floating-point implementations.

The paper is organized as follows: Section 2.1 provides details on the dataset. Section 2.2 describes the architecture of the proposed SBCNN. Section 2.3 describes the architecture of a 2T-2R OxRAM-based in-memory computing array. Section3.1 provides analysis of performance of proposed SBCNN on the CIFAR-10 and microscopy datasets, and also describes algorithm used for implementing SBCNN-based pathogen detector. Section 3.2 compares performance of implemented network across multiple computing platforms and also across memory technologies used for implementing in-memory computing. Finally, in Section 4, we summarize the conclusions of the study.

## 2. Materials and Methods

### 2.1. Dataset Description

#### 2.1.1. Automated Laboratory Diagnostics Dataset

**Figure 1:**
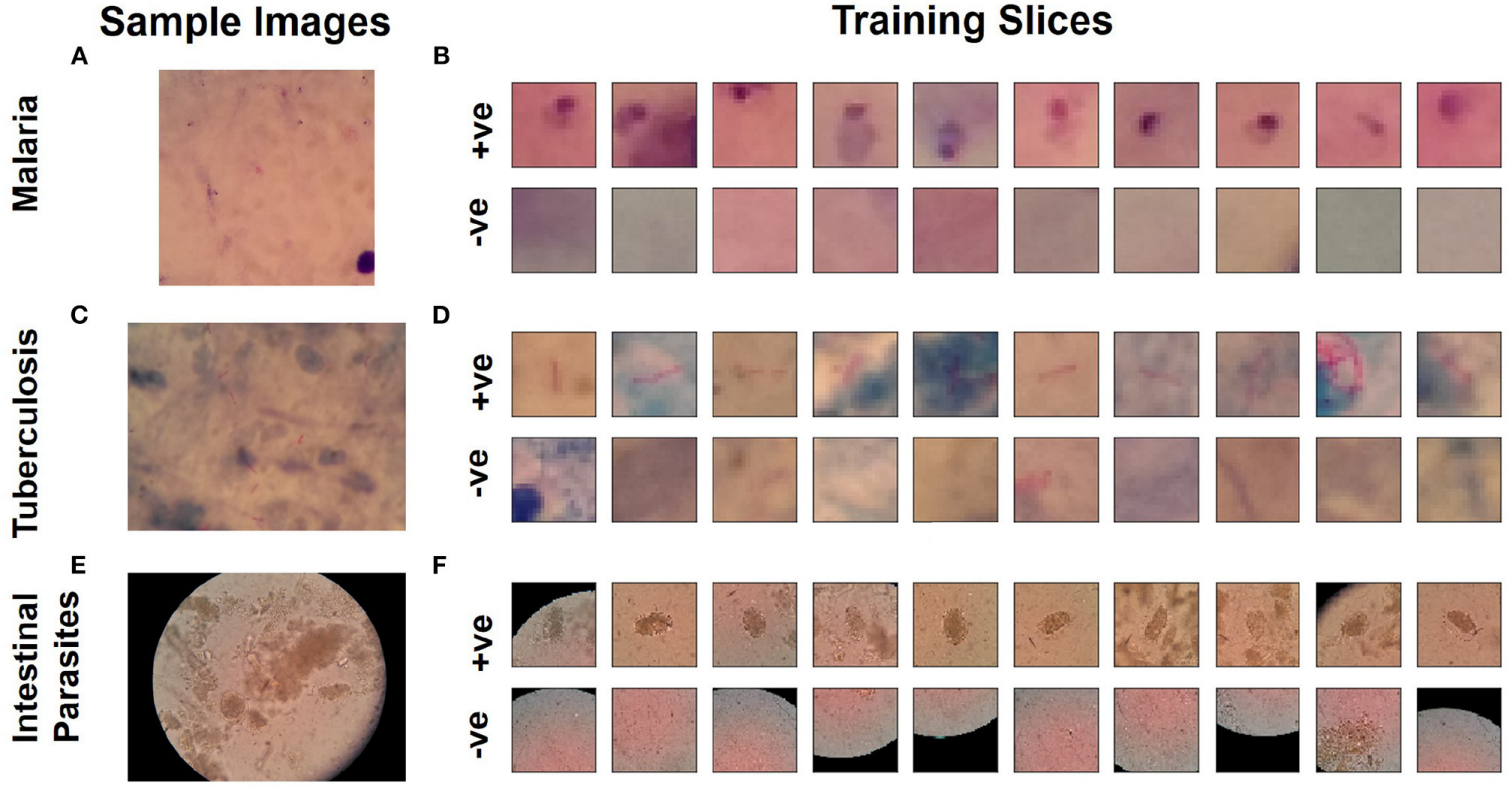
Microscopy image samples from dataset (Quinn et al., 2016) for diseases. **(A)** Malaria, **(C)** tuberculosis, and **(E)** intestinal parasite. Sample slices used for training for classification network to detect pathogen: **(B)** Malaria, **(D)** tuberculosis, and **(F)** intestinal parasite.

The automated laboratory diagnostics dataset released by the Artificial Intelligence Research Group, Makerere University, Uganda (Quinn et al., 2016) has been used for this study. The dataset incorporates images acquired using a mobile camera with a microscope for the following diseases: malaria, tuberculosis, and intestinal parasites. The malaria dataset contains images taken from thick blood smears at 1,000 × magnification, with annotated plasmodium (7,245 objects in 1,182 images). The tuberculosis dataset contains images taken from fresh sputum and stained using ZN (Ziehl Neelsen) stain, at 1,000 × magnification, with annotated tuberculosis bacilli (3,734 objects in 928 images). The intestinal parasites dataset contains images taken from slides of a portion of stool sample examined under 400x magnification annotated with eggs of hookworm, Taenia and Hymenolepsis nana (162 objects in 1,217 images) (Quinn et al., 2016). Detailed description on number of training and test samples, slice dimensions are provided in Table 1. Using these images, we produced positive and negative sample images for training a binary classification model that can detect the presence of pathogen. Positive samples (i.e., those containing plasmodium, bacilli, or parasite eggs, respectively) were produced by taking the centered bounding boxes in the annotation of the dataset. Negative samples in each image (i.e., in the absence of any of these pathogens) were taken from random locations not intersecting with any annotated bounding boxes. As dominant image areas did not contain pathogen objects, the ratio of positive to negative samples was highly skewed. Thus, some negative samples were randomly discarded and new positive samples were created by applying different transformations: rotation and flipping (Quinn et al., 2016). Example training images used as input for training the binary classifiers for each dataset are shown in Figure 1. The produced sample images were then down-sized to 20 × 20 (for malaria and tuberculosis) and 30 × 30 (for intestinal parasites).

**Table 1:** Dataset samples used for experiments.

**Dataset**	**Train samples**	**Test samples**	**Slice dimensions**	**Downsample ratio**
Malaria	289,458	290,401	40 ×40	2
Tuberculosis	78,285	80,863	20 ×20	5
Intestinal parasite	1,508	1,439	60 ×60	10

### 2.2. Proposed SBCNN Methodology

Stochastic BNN studies have been primarily limited to single channels, usually on the MNIST dataset and uniform distribution-based sampling (Lee et al., 2017; Hirtzlin et al., 2019). Adapting stochastic BNN computation for multi-channel RGB data for object detection requires optimizing the channel-specific scaling (Krizhevsky et al., 2014). We propose a novel multi-channel SBCNN architecture where a stochastic binary convolutional layer is used as input layer to the BNN. To achieve an efficient implementation, pre-processing of the RGB data is first performed using mean-sigma normalization (Krizhevsky et al., 2012):


(1) $$\begin{array}{r} X_{r,i}=\frac{X_{d,i}-\mu_{i}}{\sigma_{i}}\in \left(0,1,2\right). \end{array}$$

**Figure 2:**
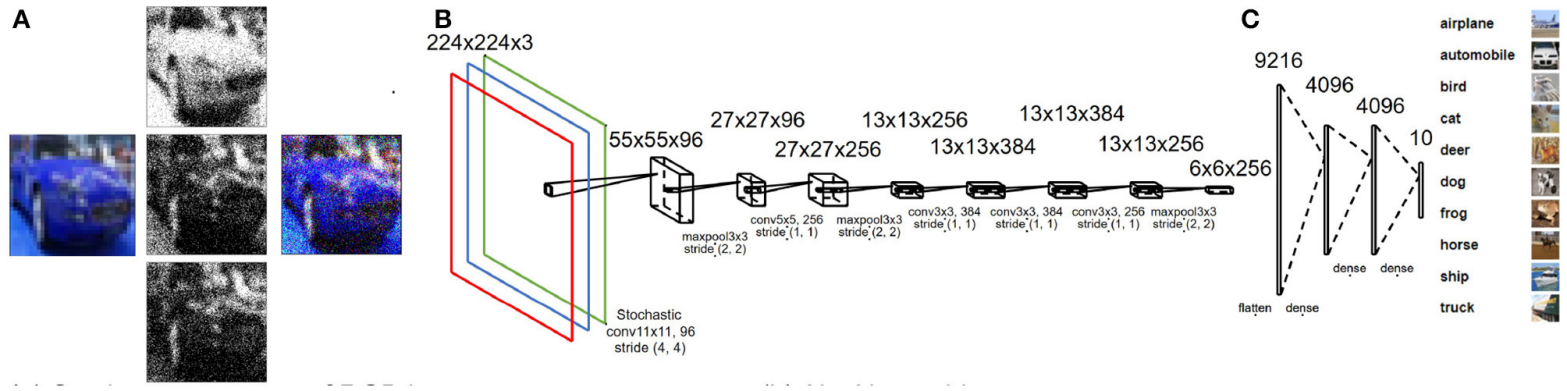
**(A)** Stochastic sampling for multi-channel input images based on normal distribution. **(B)** Modified AlexNet architecture used for CIFAR-10-based case study. **(C)** CIFAR-10 dataset samples for each class.

Here, *X*_*r, i*_ denotes the normalized response, *X*_*d, i*_ denotes the actual input, and *i* denotes the color channel. The dataflow is shown in Figure 2. This type of rescaling is often used to enhance the accuracy of deep neural networks.

Uniform distribution is generally accepted as the gold standard for implementation of stochastic computing (Alaghi and Hayes, 2013). However, capturing the response of the rescaled RGB image based on sampling using a uniform distribution may not always be efficient, as the rescaled pixels may not have an absolute minimum and maximum value. Also, as mentioned in Section 1, uniform distributions often require additional post-processing circuitry in order to be generated directly in hardware. Hence, we investigate stochastic sampling from a normal distribution based on mean-sigma normalization parameters. A single binary sample can be obtained as shown in Equation (2).


(2) $$\begin{array}{r} X_{b,i}=X_{r,i}>randn\left(\mu_{i},\sigma_{i}\right)\in \left(0,1,2\right). \end{array}$$

**Figure 3:**
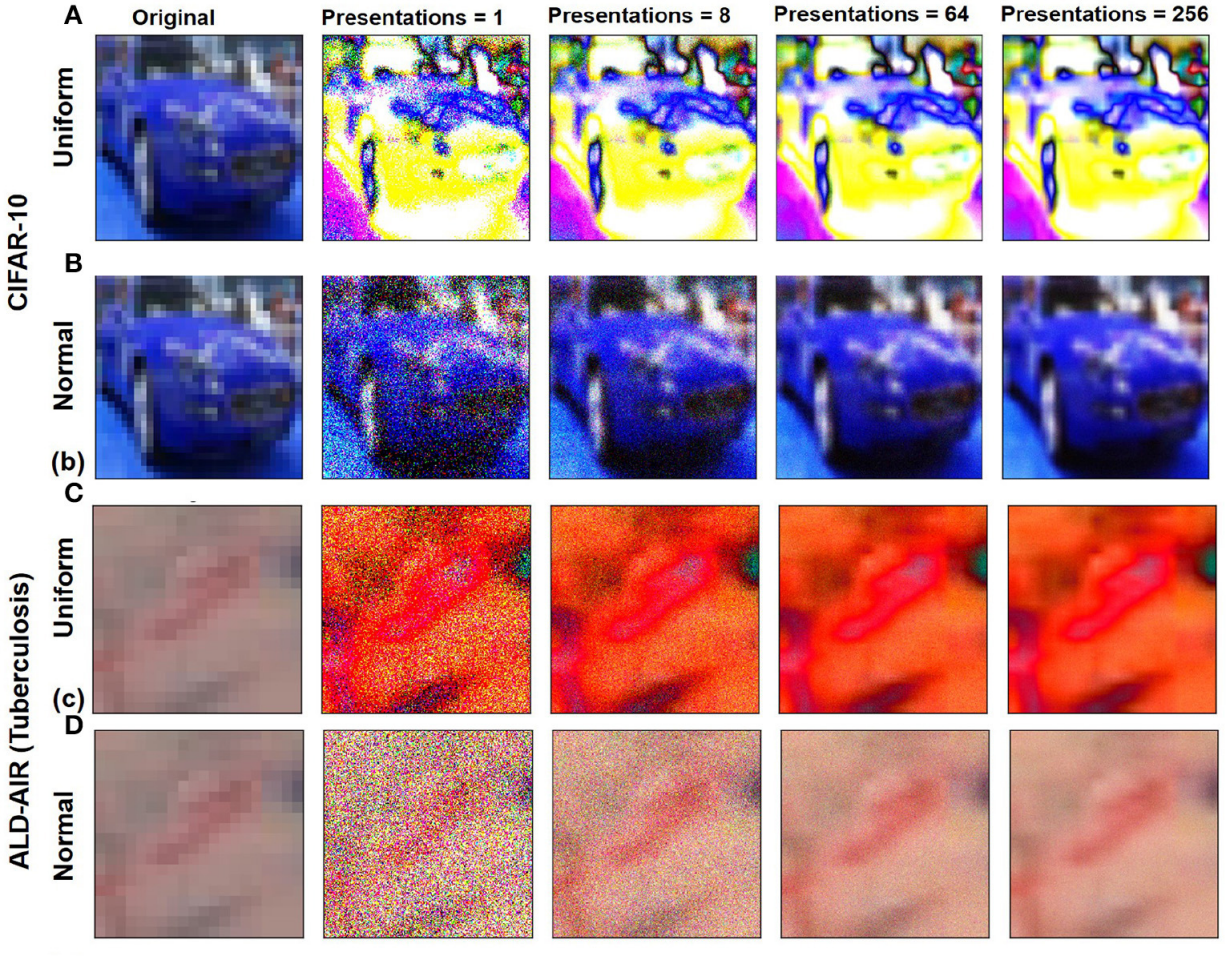
Variation in stochastic input representations based on *N*_*pre*_ and for CIFAR-10 image sample by stochastic sampling using distributions: **(A)** Uniform and **(B)** normal. Variation in stochastic input representations based on *N*_*pre*_ and for ALD-AIR image sample by stochastic sampling using distributions: **(C)** Uniform and **(D)** normal.

Here, *X*_*b, i*_, μ_*i*_, σ_*i*_ denote binary sample value, mean, and standard deviation for a pixel in channel *i*, respectively, while *randn* is a random number obtained using a normal distribution. For a single pixel, samples are collected as a stream of binary values by repeated sampling for *N*_*pre*_ presentations to better capture the complete input range (shown in Figure 3):


(3) $$X_{m,i}=\frac{\sum_{j=1}^{N_{pre}}x_{b,i}}{N_{pre}}.$$

Here, *X*_*m, i*_ denotes summation of the stochastic samples stream.

**Figure 4:**
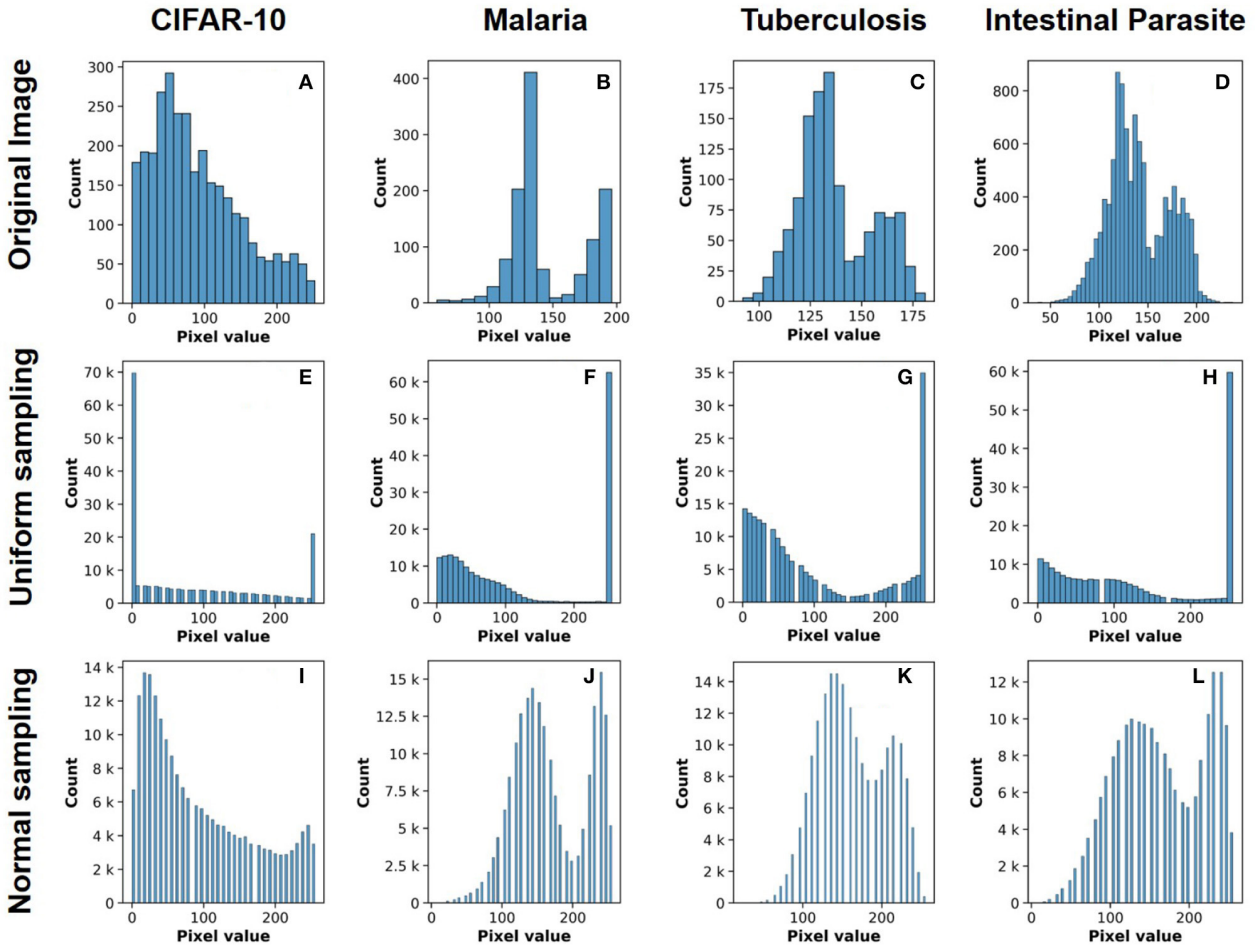
Histograms of pixel-value distribution over image samples from the datasets used in the study: **(A–D)** original image, **(E–H)** uniform distribution based stochastic sampling, and **(I–L)** normal distribution-based stochastic sampling.

The network architecture used throughout our study is based on the AlexNet architecture (Krizhevsky et al., 2012) with the output layer restricted to 10 neurons (multi-class classification) for CIFAR-10 (Figure 2) and two neurons (binary classification) for ALD-AIR dataset. The neural networks used in the study were trained as per the method proposed by *et al*. (Courbariaux et al., 2016). Both quantized and binarized networks were explored to estimate the impact of precision. The Adam optimizer (Kingma and Ba, 2014) was used for optimizing the loss during training. To introduce stochastic computing in the network, we build upon the method proposed by (Hirtzlin et al., 2019). Representations of sample input images based on stochastic presentations using uniform and normal distribution-based sampling are shown in Figure 3 for both CIFAR-10 and ALD-AIR datasets. Histograms of pixel-wise intensity across all 3 channels for sample images from each dataset are shown in Figure 4.

**Table:** 

**Algorithm 1 |** SBCNN inference algorithm.
**Require:** Input vector X, Weight matrices *W*_*n*_, Bias vectors *b*_*n*_, #Layers L #Presentations N.
**Ensure:** Predicted output
** Stochastic Layer**
*a*_*n*_ = sign(popcount(XNOR(*W*_0_,*X*>*rand*)) - *b*_0_)
*A*_0_ = sign($\overset{N}{\underset{n=0}{\sum }}a_{n}$)
**Regular Layer**
**for** *i* = 1;*i*<*L*; *i* = *i*++ **do**
*A*_*i*_ = sign(popcount(XNOR(*W*_*i*_,*A*_*i*−1_)) - *b*_*i*_)
**end for**

### 2.3. Hardware Implementation Based on Emerging Memory Devices

The major hardware realizations for implementing proposed SBCNN include stochastic sampling at input layer and computation of BNN. In BNNs, weight values are one-bit (weight can only take values −1 and +1), and neuron activation is implemented by the sign function. Neuron output is computed by


(4) $$\begin{array}{r} y=\text{sign}\left(\text{popcount}\left(\text{XNOR}\left(w_{j},x_{j}\right)\right)-b\right). \end{array}$$

Here, popcount is a function counting the number of ones, and *b* is a learned neuron's threshold. Besides reducing memory requirements due to reduced precision, BNNs enable reduction of computation logic circuit area, as digital multipliers can be replaced by simple XNOR logic gates.

**Figure 5:**
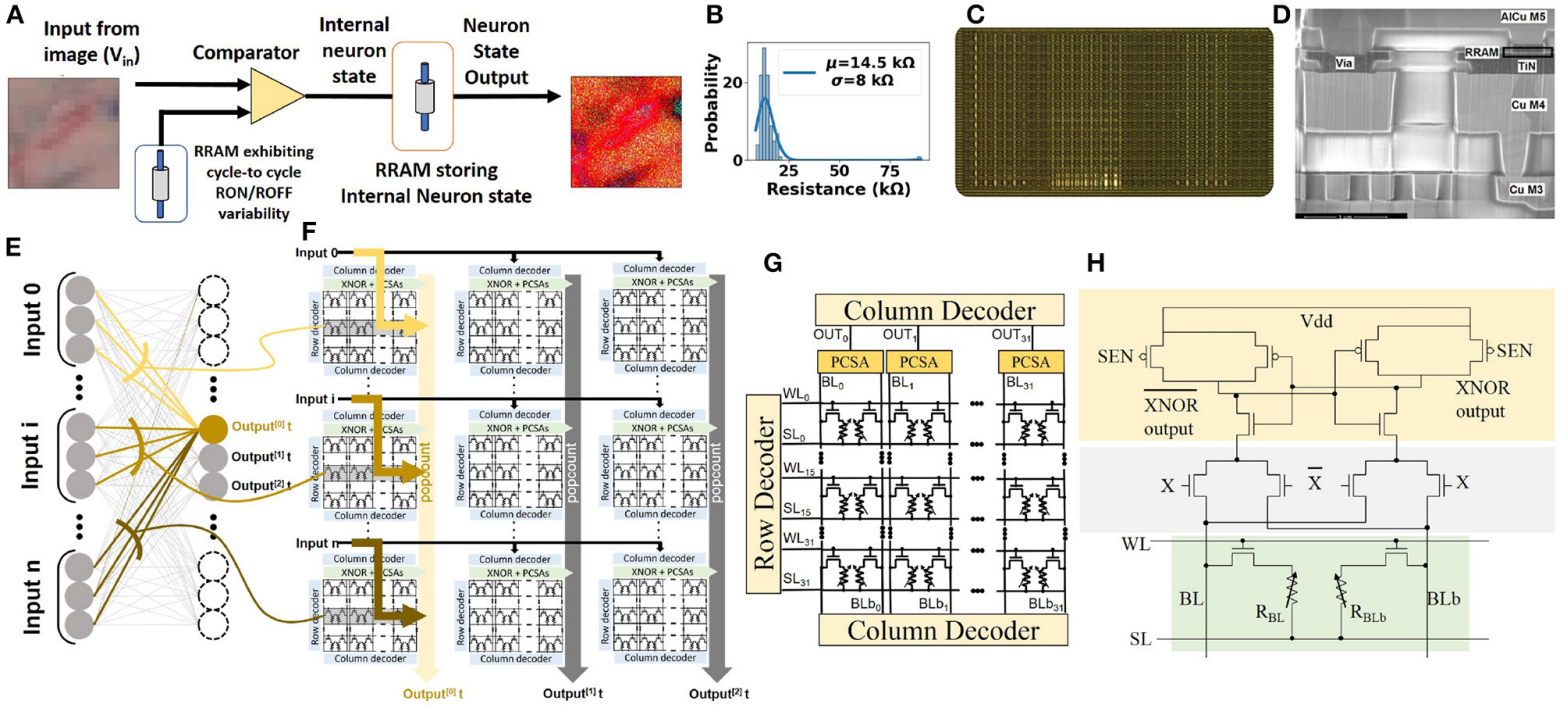
**(A)** Stochastic neuron circuit based on OxRAM device used for input sampling. 2T-2R in-memory XNOR circuit for BNN computation. **(B)** C2C variability observed in low resistance state (LRS) for the fabricated OxRAM device of (Dalgaty et al., 2021). **(C)** Memory array chip photograph. **(D)** OxRAM cell. **(E)** Binarized neural network implementation highlighting connections to one specific neuron. **(F)** Implementation of binarized neural network in the “parallel to sequential” configuration. **(G)** 2T-2R bitcell array. **(H)** Schematic of 2T-2R bit-cell for XNOR operation computation based on pre-charge sense amplifiers (PCSAs) (Hirtzlin et al., 2019).

For realizing such computation in using emerging memory devices in hardware, we introduced in previous studies (Bocquet et al., 2018; Hirtzlin et al., 2020) a hybrid CMOS/OxRAM test chip, where synaptic weights are stored in OxRAM (shown in Figure 5D), and which utilizes the 2T-2R architecture (shown in Figures 5A,C) to store synaptic weights in a differential fashion: a device pair programmed in low resistance state (LRS)/high resistance state (HRS) represents +1, and, conversely, HRS/LRS represents −1. Pre-charge sense amplifiers (PCSAs) compare the resistance states of the two paired devices, thus reading the synaptic weight. An advantage of this approach is the possibility of incorporating the XNOR operation utilized in BNN computation directly within PCSA by the addition of four transistors (shown in Figure 5H). Figures 5E,F presents the methodology for implementing fully connected layers, by minimizing data movement (Hirtzlin et al., 2019). Training is performed off-chip, followed by weight programming and inference operations. We use this implementation of BNN as a reference in this work.

Emerging memory devices such as OxRAM devices have been shown to demonstrate normal C2C *R*_*OFF*_ distribution, which has been exploited for stochastic sampling applications (Suri et al., 2015; Dalgaty et al., 2021). A circuit implementation for the same is shown in Figure 5A. Each stochastic neuron accepts an image pixel in form of voltage encoding that is compared with the voltage drop across the OxRAM device, which is repeatedly cycled from LRS to HRS. The intrinsic C2C *R*_*ON*_ variability of the OxRAM device leads to a variable reference voltage for the comparator. LRS variability of a fabricated OxRAM device (Dalgaty et al., 2021) is shown in Figure 5B. This enables translation of deterministic input voltage to a stochastic binary neuron output.

## 3. Results and Discussions

### 3.1. Simulation Results and Discussion

#### 3.1.1. Case study A: CIFAR-10

To evaluate the proposed SBCNN for generic image classification applications, we performed analysis using the CIFAR-10 dataset (Krizhevsky et al., 2014). The *N*_*pre*_ parameter used for training was 32. A benchmarking of the proposed SBCNN is shown in Table 2. For all stochastic computations, average results obtained over five trials are listed.

**Table 2:** Test accuracy benchmarking of different precision networks, simulated in this study, for CIFAR-10 dataset.

**Network description**	**Input layer precision**	**Test accuracy (%) (Top-1)**
AlexNet (FP-32 precision)	FP-32	88.64
AlexNet (INT8 precision)	INT8	87.57
AlexNet (BNN)	INT8	86.92
SBCNN (uniform) (*N*_*pre*_ = 32)	Binary	82.89
^*^SBCNN (normal) (*N*_*pre*_ = 32)	Binary	85.61

The performance of our proposed method based on sampling from normal distribution matches AlexNet closely (≈3%) even using 32-bit floating point precision (FP32). In contrast, the network based on sampling from uniform distribution results in a higher accuracy drop (≈6%).

*N*_*pre*_ is an important parameter to realize equivalent accuracy with a reduced number of operations. To understand the impact of this parameter, we also analyzed two strategies:

- *Max presentations:* We train the network with a maximum number of presentations (256) and infer with *N*_*pre*_ presentations.
- *Matched presentations:* Training and inference are performed using the same *N*_*pre*_ number of presentations.

**Figure 6:**
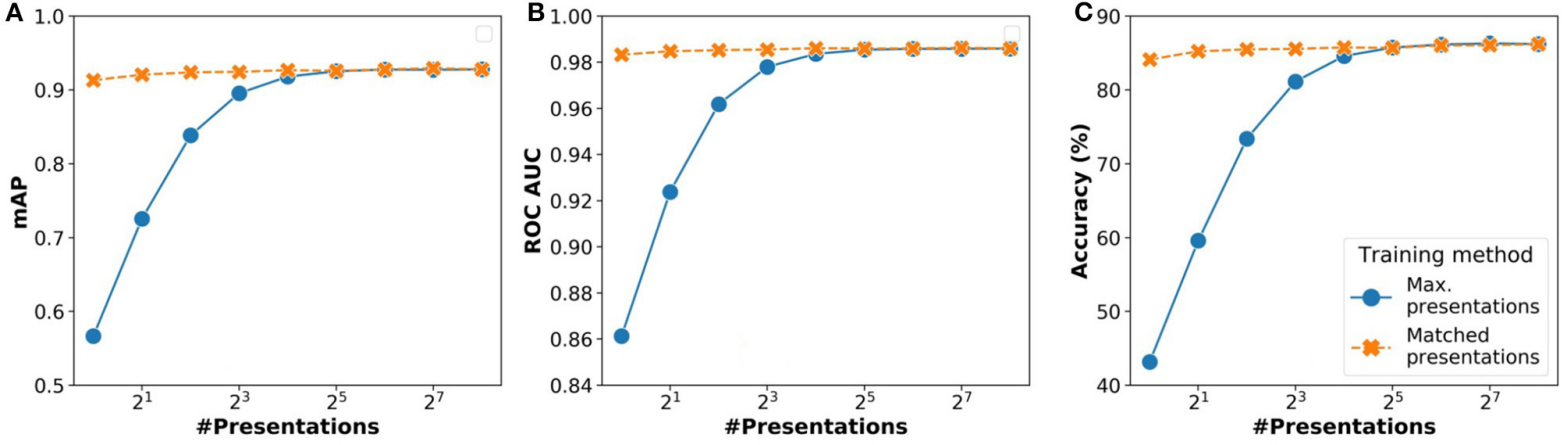
Variation of network performance metrics with *N*_*pre*_ for inference using stochastic binarized neural network (BNN) (AlexNet model) for CIFAR-10 dataset: **(A)** Accuracy, **(B)** mean average precision, and **(C)** receiver operating characteristics area under curve. The results have been averaged over 5 iterations.

Results of the analysis comparing these two strategies are shown in Figures 6A–C. We analyzed the overall impact on inference in terms of three parameters: (i) accuracy (%), (ii) mAP (mean average precision), (iii) ROC AUC (receiver operating characteristics area under curve). For all three parameters, the performance of the matched presentations method is observed to be consistently better than the max. presentations method. The matched presentation method leads to accuracy values that are close to *N*_*pre*_ = 256 for all values of *N*_*pre*_. We also observed that using matched presentation method with *N*_*pre*_ = 8 showed equivalent accuracy as the max. presentations case for *N*_*pre*_ = 256. The matched presentation method, therefore, appears vastly superior.

#### 3.1.2. Case Study B: Microscopy

Point-of-Care (PoC) microscopy diagnostic support systems for different diseases (e.g., malaria, tuberculosis, and intestinal parasite infection) have been studied in detail with regard to application of deep learning. However, most of the implementations in the literature are based on conventional CPUs (Yang et al., 2020), high-end GPUs (Quinn et al., 2016), or FPGAs (Yokota et al., 2002; Grull et al., 2011). Recent work has also explored possibility of realizing such PoC systems using specialized ASIC accelerators with reduced energy consumption (Sethi et al., 2018). Here, we present a case-study for the application of the proposed SBCNN for microscopy image analysis for potential application specific optimization with the goal of low-power/low-resource edge realizations.

**Figure 7:**
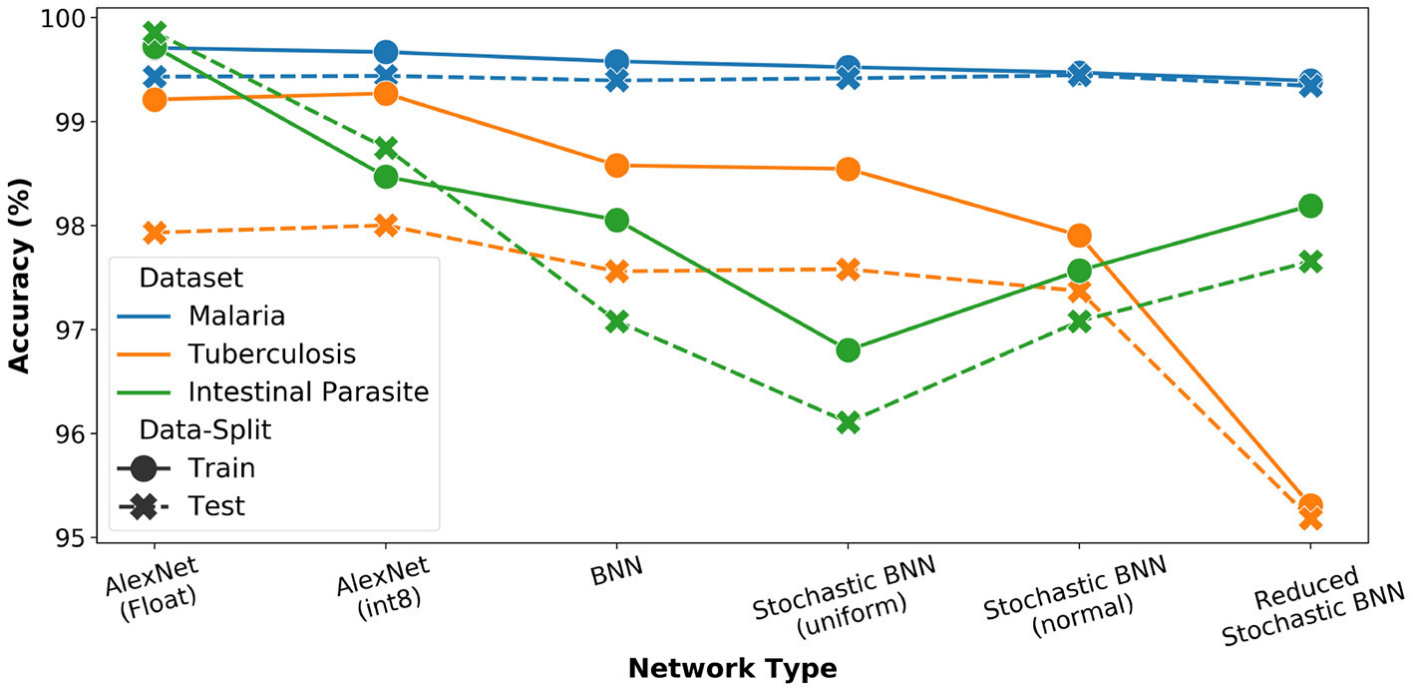
Network precision and architecture analysis for microscopy diagnosis task. For all stochastic networks, training and inference is performed with *N*_*pre*_ = 32.

The AlexNet architecture was again used as reference model for the study (Krizhevsky et al., 2012). We performed analysis for *FP-32, int8*, as well as binary precisions and plotted the results of both training as well as inference accuracy in Figure 7. We observe the highest accuracy for FP-32 and a consistent accuracy reduction when moving to lower precision.

Further, we estimated performance for SBCNN with both uniform and normal distribution-based sampling (*N*_*pre*_ = 32). The results are also reported in Figure 7. We observe increased or equivalent accuracy when transitioning from uniform to normal distribution-based sampling. To further optimize the network architecture given the lower complexity of the task (i.e., binary classification), we reduced the network depth by removing an intermediate linear layer leading to increased memory savings. This modified network architecture is referred to as reduced SBCNN. As observed in Figure 7, the total accuracy drop between best-case FP32 and the optimized reduced SBCNN is approximately 5%. Furthermore, we can also observe that the impact of bit-precision trade-off with accuracy is more pronounced for datasets with less training data (malaria vs. tuberculosis). In case of intestinal parasites dataset, this trend is reversed due to the small size of the dataset resulting in overfitting, even with reduced precision.

**Figure 8:**
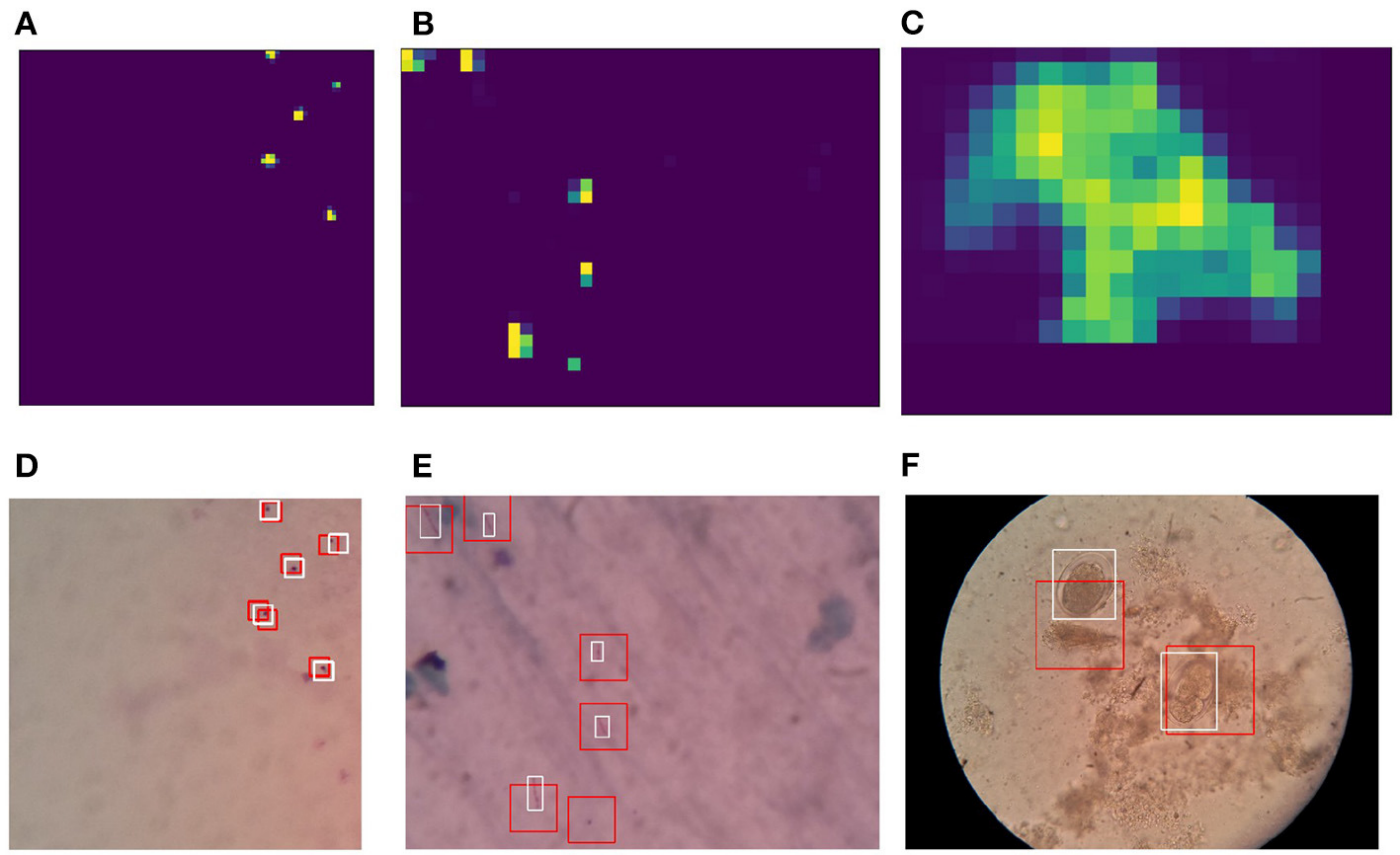
Heatmaps generated based on sliding window-based detections using reduced stochastic binarized neural network (BNN) classifiers: **(A)** Malaria, **(B)** tuberculosis, and **(C)** intestinal parasite. Detections on microscopy sample images: **(D)** Malaria, **(E)** tuberculosis, and **(F)** intestinal parasite. Red box indicates network-based detection, and white box indicates ground truth. The *N*_*pre*_ used for training and inference are 32.

After the training step described in Section 2.2, the classifiers can be used for performing detection of pathogens using the sliding window approach on microscopy images. As part of the sliding window approach, the classifier output is summed over the pixels of the window in order to generate a heat map. The sliding windows have 50% overlap in both horizontal and vertical directions. Heat map outputs generated based on the sliding windows are then normalized, followed by a threshold operation in order to generate candidate regions in form of binary maps. Bounding boxes are generated based on contour detection performed over these binary maps. To improve detection quality, non-maximal suppression was applied. Results for the detection for each type of pathogen with corresponding heatmaps are shown in Figure 8.

#### 3.1.3. Learning Performance

We characterized the accuracy of all network architectures using two metrics: ROC curve and precision-recall (PR) curve. ROC curves are used to visualize the precision capacity of the network by plotting TPR (true positive rate) and FPR (false positive rate) as functions of threshold. A steep slope and concentration near one demonstrate very high precision and, in turn, less chances of false positives. For ROC curves, the AUC is measured as a performance parameter. AUC equal to one is typically observed for an ideal classifier, whereas AUC equal to 0.5 is observed for classifiers with the worst performance (Hajian-Tilaki, 2013).

While such estimates for identifying positives are important, it is also necessary to understand impact of false negatives. Hence, PR curves are used. PR curves show the trade-off between precision (1-FDR, where FDR means False Discovery Rate) and recall (TPR). In case of an ideal curve, the precision remains unchanged and at maximum until recall reaches one. This curve also forms the basis for estimating mean average precision (mAP).

**Figure 9:**
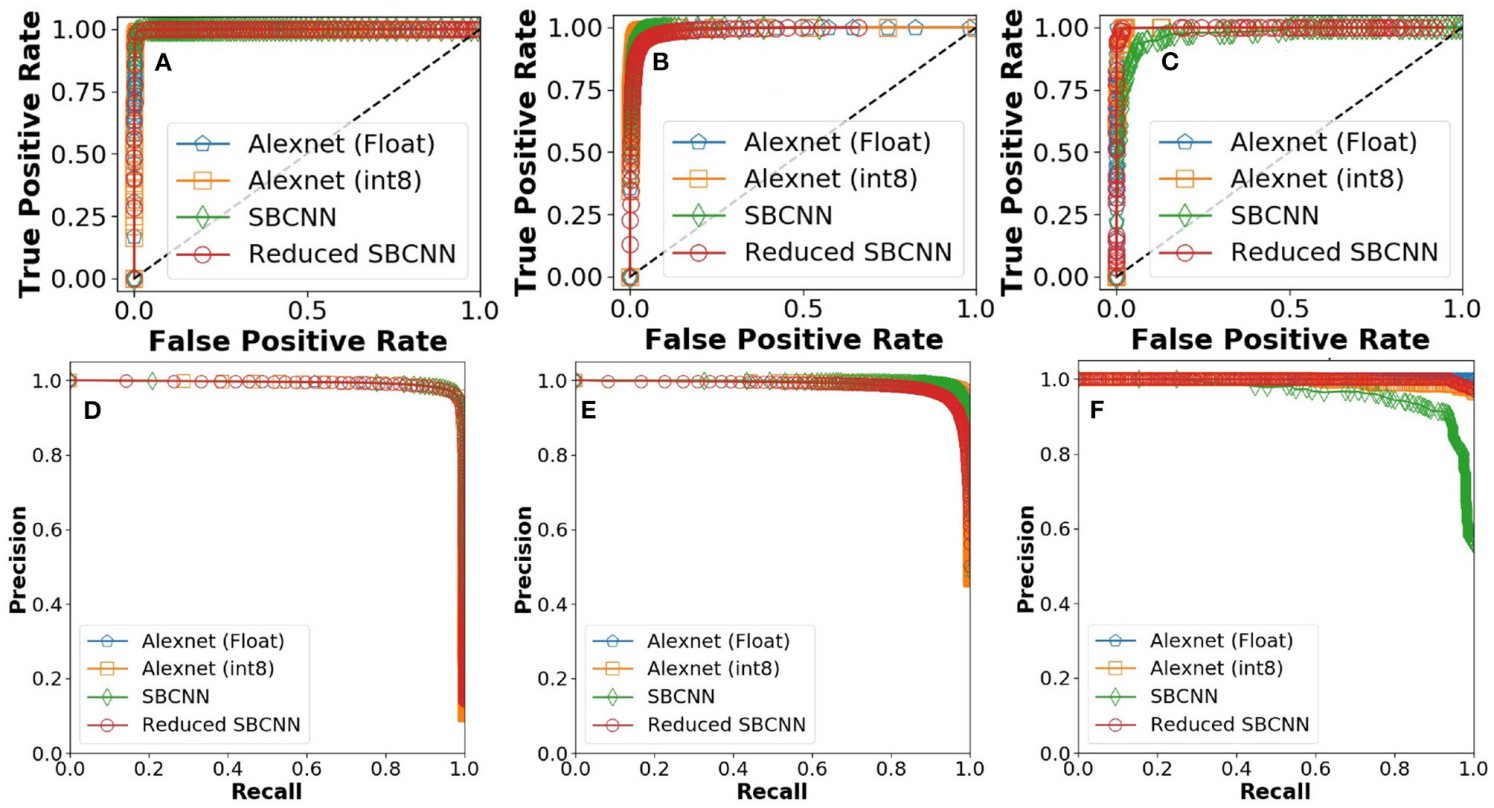
Receiver operating characteristics (ROC) curves for proposed stochastic binarized convolutional neural network (SBCNN):(normal distribution and reduced layers): **(A)** Malaria, **(B)** tuberculosis, and **(C)** intestinal parasite. Precision recall curves for proposed SBCNN (normal distribution and reduced layers): **(D)** Malaria, **(E)** tuberculosis, and **(F)** intestinal parasite.

ROC and PR curves for the experiments were calculated by averaging performance parameters over five iterations for stochastic networks. As shown in Figure 9, the smallest network architecture is able to match the learning performance of FP-32 AlexNet.

#### 3.1.4. Impact of Stochastic Presentations

**Figure 10:**
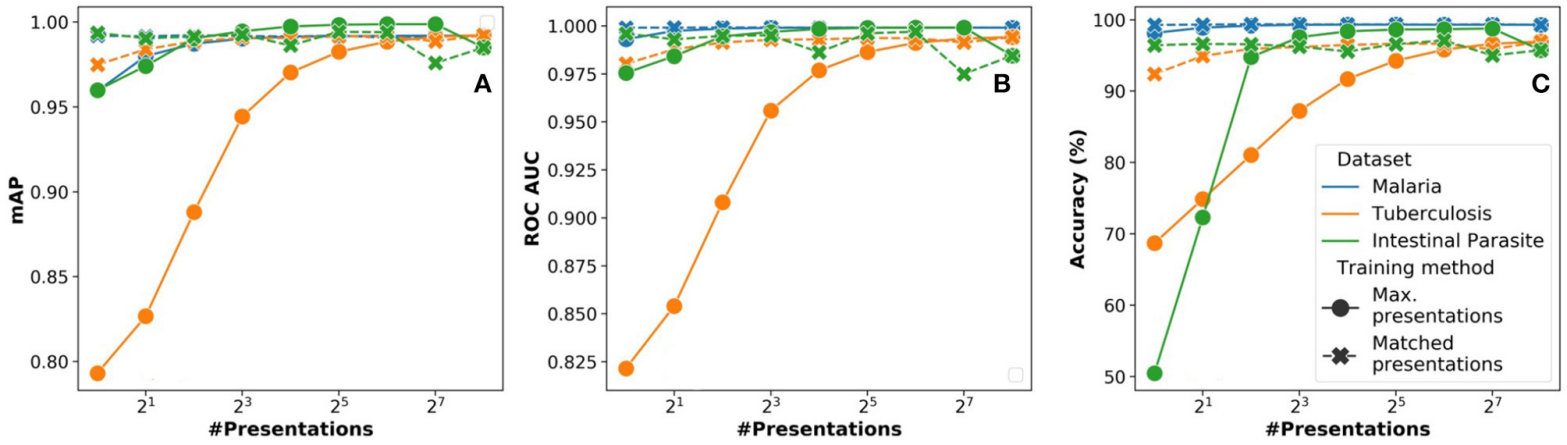
Variation of network performance metrics with *N*_*pre*_ for stochastic binarized convolutional neural network (SBCNN) (reduced model) on microscopy diagnosis task: **(A)** Accuracy, **(B)** mean average precision (mAP), and **(C)** ROC_AUC. Results have been averaged over 5 iterations.

In Figure 10, we analyze the impact of the *N*_*pre*_ parameter used during inference on the overall learning performance in terms of (a) accuracy, (b) mAP, and (c) ROC AUC. When using the presentations strategy, there is a minor trade-off in overall learning performance (≈2%). The impact is more severe in case of the smallest dataset (intestinal parasites), which would result in over-fitting. When using the max. presentations strategy, we observe an increasing trend in learning performance as the *N*_*pre*_ approach the value used for training.

From the analysis, we conclude that, for a practical implementation, *N*_*pre*_ = 8 would be sufficient. As can be observed from Figure 7, training accuracy for all datasets is relatively constant (≤ 4% difference) for all architectures. However, there is a major trade-off in computation complexity and memory requirement as shown in Table 3.

**Table 3:** Performance estimates of inference with multiple architectures for microscopy image analysis.

**Network type**	**Platform**	**Weight memory (MB)**	**MAC ops (Mops)**	**Energy (μJ)**
			**per inference**	
AlexNet (Float)	GPU (RTX 2080)	217.42		1.47e5
AlexNet (int8)	ASIC (Eyeriss v2)	54.37		5.72e3
	(Chen et al., 2019)			
BNN	2T-2R IMC	7.53	15.24	7.98
Stochastic BNN		7.53	12.91	6.84
Reduced model		4.79	10.74	5.66

### 3.2. Performance Analysis: Memory, Energy, and Delay

To compare the different architectures proposed in the study, we estimated the number of operations and energy corresponding to each network architecture for mapping them on the 2T-2R OxRAM XNOR bitcell array. As shown in Figure 5E, multiple kilobit arrays of 2T-2R cells can be arranged in a matrix structure in order to allow parallel computation. We assume a 32 × 32 matrix of tiles of 32 × 32 2T-2R bitcell arrays (shown in Figure 5G). Weight mapping is performed with respect to the block matrix multiplication with the division of weights having a block size of 32. For computation within each block, it is assumed that only a single row can be computed in each cycle, thus requiring 32 cycles for completing computation across the complete block. Therefore, only a single five-bit look-up table would be required for each block, leading to lower area utilization. For performing energy estimations for *float* and *int8* precisions, an Nvidia Turing GPU-enabled server and the Eyeriss v2 chip (Chen et al., 2019) are used as reference platforms. These two platforms store synaptic weights in off-chip dynamic RAM. Furthermore, for stochastic computing implementation, we assume that each input is sampled simultaneously from the stochastic circuit shown in Figure 5A. Results comparing implementations of networks with varying bit precisions are described in Table 3. As shown in Table 3, converting a conventional accelerator-based 8-bit computation to stochastic binarized in-memory computation with *N*_*pre*_ = 32 results in savings of ≈1,000 × in energy and ≈11 × in memory. Reduced version of the SBCNN offers savings of 36% in memory and 17% in energy, while still maintaining comparable accuracy. A comparison of the proposed hardware with regard to other techniques of the literature for implementing BNN hardware is shown in Table 4.

**Table 4:** Benchmarking performance with respect to other literature studies for implementation of binarized AlexNet.

**Technology**	**Energy/frame (μJ)**	**References**
DRAM	72,833.21	Li et al., 2017
	3,427.83	Sudarshan et al., 2019
	660.00	Jiang et al., 2017
SRAM	23.30	Yin et al., 2020
SOT-MRAM	561.30	Angizi et al., 2019
	310.00	Fan and Angizi, 2017
OxRAM	2275.34	Tang et al., 2017
	**5.66**	^*^This work

*The value in bold refers to estimated value from the current work*.

## 4. Conclusion

In this study, we proposed a hardware-friendly stochastic binarized convolutional neural network architecture for performing energy-efficient near-sensor computing, using stochastic sampling from non-uniform distributions. We first validated the proposed implementation using the CIFAR-10 dataset for generic classification applications. Next, we investigated a case study for microscopy-based pathogen detection. Accuracy of the optimized network proposed in the study is similar to previous works with floating-point precision but exhibits memory savings in the order of ≈45 ×. We further analyzed the benefits of realizing such networks using in-memory computing based on emerging non-volatile memory devices. We studied in detail the impact on network performance in terms of accuracy, energy due to levels of quantization and network architecture changes. The proposed architecture shows up to ≈1,000 × reduction in energy and weight memory savings of ≈ 11 × compared to the standard architectures. An end-to-end methodology from training algorithm to dedicated hardware implementation is also discussed.

## Data Availability Statement

The datasets analyzed for this study can be found in the following links: (i) CIFAR-10: https://www.cs.toronto.edu/~kriz/cifar.html (ii) Automated Laboratory Diagnostics Dataset: http://air.ug/microscopy/.

## Author Contributions

VP and BP performed the BNN simulations. MS and DQ planned and supervised the project. All authors participated in data analysis and writing of the manuscript.

## Funding

This work was supported by DST SERB CORE Research grant (CRG/2018/001901), IIT-D FIRP grant, CNRS-PICS, the European Research Council grant NANOINFER (grant no. 715872).

## Conflict of Interest

The authors declare that the research was conducted in the absence of any commercial or financial relationships that could be construed as a potential conflict of interest.

## Publisher's Note

All claims expressed in this article are solely those of the authors and do not necessarily represent those of their affiliated organizations, or those of the publisher, the editors and the reviewers. Any product that may be evaluated in this article, or claim that may be made by its manufacturer, is not guaranteed or endorsed by the publisher.
